# Strain Transfer Analysis of Rubber-Encapsulated Fiber Bragg Grating Sensors for Wind Turbine Blade Strain Monitoring

**DOI:** 10.3390/mi17070784

**Published:** 2026-06-27

**Authors:** Jianping He, Zhilong Zhou, Tongchun Qin, Qiyu Qu, Jiangpei Zhu

**Affiliations:** 1 School of Civil Engineering, Nantong Institute of Technology, Nantong 226000, China; 2 School of Information Engineering, Nantong Institute of Technology, Nantong 226000, China

**Keywords:** rubber-encapsulated sensor, FBG, strain transfer, wind turbine blade, EPDM rubber, experimental verification, FBG strain rosette

## Abstract

To resolve the discrepancy between the measured strain and the actual surface strain of wind turbine blades when using rubber-encapsulated fiber Bragg grating (FBG) sensors for strain monitoring, this study establishes a surface-bonded strain transfer model for such sensors. The total strain transfer efficiency of the sensor is decomposed into two components: the strain transfer efficiency from the rubber substrate to the FBG core (encapsulated grating strain transfer efficiency) and that from the wind turbine blade to the rubber substrate (strain transfer efficiency between the rubber substrate and the blade). Based on the theory of mechanics of materials, the strain transfer equation is derived, and the key factors influencing strain transfer efficiency—FBG bonding length and rubber substrate thickness—are analyzed via the control variable method. Three ethylene propylene diene monomer (EPDM)-encapsulated FBG sensors with rubber substrate thicknesses of 3 mm, 4 mm, and 6 mm were fabricated. Tensile strain transfer tests were conducted using fiber-reinforced plastic (FRP) strips to simulate the material properties of wind turbine blades, so as to validate the effectiveness of the proposed model. The experimental results demonstrate that the strain transfer efficiency of the sensor increases with the extension of FBG bonding length and decreases with the increase in rubber substrate thickness, with 4 mm determined as the optimal substrate thickness for EPDM-encapsulated FBG sensors. On the basis of the aforementioned findings, an EPDM-encapsulated FBG strain rosette sensor was developed, which can effectively measure the complex stress of a wind turbine blade model. This study provides a theoretical foundation for the structural design and engineering application of rubber-encapsulated FBG sensors in the strain monitoring of wind turbine blades.

## 1. Introduction

Wind turbine blades are the core components of wind turbines for wind energy capture. They operate long-term under complex and harsh service conditions, including alternating wind loads, temperature fluctuations, ultraviolet radiation and environmental corrosion, and are prone to structural damage. Therefore, accurate monitoring of the structural strain state of wind turbine blades is essential to ensure the safe and stable operation of wind turbines, predict structural fatigue life, and prevent sudden structural failure. At present, apart from non-destructive testing techniques including unmanned aerial vehicles (UAVs) and light detection and ranging (LiDAR), the most straightforward method is to install strain sensors on wind turbine blades, so as to obtain the real-time stress–strain behavior during their service [1,2,3,4]. In terms of blade strain monitoring technology, strain measurement methods such as resistance strain gauges and piezoelectric films have the advantages of simple operation, low cost, and high accuracy. However, their measurement accuracy is easily affected by temperature, electromagnetic interference, and thunderstorm disturbances. Moreover, these methods only support single-point measurement, making them difficult to adapt to the complex and harsh operating environment of wind turbine blades and the engineering requirements of large-scale strain monitoring [5,6,7]. In contrast, optical fiber sensing technology, including fiber Bragg grating sensing technology and distributed optical fiber sensing technologies, offers prominent advantages including electromagnetic immunity (EMI), high measurement accuracy, non-conductivity, long service life, and the capability to realize distributed and quasi-distributed monitoring, and has been successfully applied in fields such as bridges, tunnels, and roadbeds [8,9,10]. For wind turbine blade monitoring, FBG, weak FBG, distributed optical fiber Brillouin sensing technology and OFDR technology have also been applied in both wind turbine blade model tests and on-site monitoring [11,12,13]. For examples, Wang et al. established the theoretical model for structural measurement of FBG-based wind turbine monitoring sensors and designed the sensor structure, analyzed the sensor layout positions and data processing methods during blade strain measurement, and provided a theoretical basis for wind turbine blade health monitoring using adhesive FBG strain sensors [14]; Hsu et al. proposed a damage detection approach based on dynamic macro-strain signals from long-gauge FBG sensors, which can effectively obtain the modal parameters of rotating wind turbines and locate the stiffness variation regions of wind turbine blades [15]. Lu et al. carried out wind turbine blade monitoring based on OFDR technology, investigated the strain distribution law of full-scale healthy/cracked wind turbine blades under steady-state wind loads, and analyzed the blade strain characteristics under dynamic conditions [16].

In the above studies, bare optical fibers or bare FBGs were directly pasted on the blade surfaces as sensing elements in small-scale wind turbine blade model tests. Bare optical fibers and FBGs are slender in size and possess poor shear resistance and impact resistance, making them unsuitable for on-site application on wind turbine blades. Therefore, encapsulated FBG sensors or distributed optical fiber strain sensors are commonly adopted for blade monitoring in practical engineering. The selection of encapsulation materials directly affects the protective effect and strain transfer performance of the sensors. Accordingly, numerous scholars have investigated the strain transfer characteristics of encapsulated sensors and their testing efficiency after layout and installation on monitored components [17,18]. For example, Wang et al. proposed the strain transfer models under thermal, static and dynamic loading conditions, and verified the effectiveness and accuracy by lab tests, which can be adopted to instruct the sensor design and parametric reflection in practical engineering [19,20]. Wind turbine blades are mostly composite structures, and the application of conventional metallically packaged sensors on them presents two main problems: first, metallically packaged sensors are prone to corrosion and have poor durability; second, their high rigidity makes them unsuitable for monitoring the large deformation of wind turbine blades, and they are prone to debonding from the blade surface during service. Rubber materials possess excellent elasticity, weather resistance, aging resistance, and flexibility. They can effectively protect FBG sensors from external damage while adapting to the flexible deformation of wind turbine blades, making them ideal encapsulation materials for FBG sensors used in wind turbine blade strain monitoring. Despite the significant advantages of rubber encapsulation, the rubber layer prevents the FBG sensor from making direct contact with the blade surface. During the strain transfer process, shear deformation and elastic modulus mismatch between the encapsulation layer, adhesive layer, and blade surface lead to strain transfer loss, resulting in a discrepancy between the sensor-measured strain and the actual surface strain of the wind turbine blade. This discrepancy reduces the reliability of monitoring data and may even lead to misjudgment of the blade’s structural state, seriously affecting the accuracy of blade structural health assessment. Therefore, in-depth research on the strain transfer characteristics of rubber-encapsulated FBG sensors is urgently needed.

In view of this, this paper takes ethylene propylene diene monomer (EPDM) rubber-encapsulated FBG sensors as the research object, establishes a surface-bonded strain transfer model suitable for strain monitoring of wind turbine blades, and derives the strain transfer equation in detail based on the theory of mechanics of materials. Meanwhile, an EPDM rubber-encapsulated FBG strain rosette sensor is developed to conduct research on complex stress monitoring of blade models. The research results can provide an important theoretical basis and technical support for the structural optimization design and engineering application of rubber-encapsulated FBG sensors in wind turbine blade strain monitoring, and promote the development of wind turbine blade strain monitoring technology toward higher precision and reliability.

## 2. Principle of Strain Transfer Analysis

**Figure 1:**
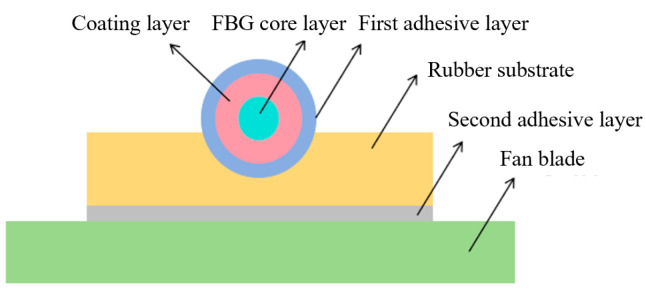
Cross-sectional view of the sensor.

When a rubber-encapsulated FBG sensor is bonded to the surface of a wind turbine blade for strain monitoring, its structural layers from the inside to the outside include the FBG core layer, coating layer, first adhesive layer, rubber substrate, second adhesive layer, and the wind turbine blade being measured, as shown in Figure 1. In practical applications, the rubber protective sheet only plays a physical protective role, and its influence on the strain transfer efficiency is negligible. The strain transfer process of the sensor can be divided into two independent stages: the first stage involves strain transfer from the rubber substrate to the FBG core layer through the first adhesive layer and coating layer; the second stage involves strain transfer from the wind turbine blade to the rubber substrate through the second adhesive layer. Based on this, the total strain transfer rate γ of the rubber-encapsulated FBG sensor is decomposed into the strain transfer rate α of the encapsulated FBG and the strain transfer rate β between the rubber substrate and the wind turbine blade. The strain transfer rate α is the ratio of the FBG core layer strain to the rubber substrate strain, reflecting the efficiency of strain transmission from the rubber substrate to the FBG, and the strain transfer rate β is the ratio of the rubber substrate strain to the wind turbine blade surface strain, reflecting the efficiency of strain transmission from the wind turbine blade to the rubber substrate. The theoretical relationship of the total strain transfer rate is:

(1) γ=α×β

The present strain transfer formulation follows the classical Ansari and Libo approach [21] but introduces two important modifications. (i) Ansari and Libo used a hyperbolic-sine (sinh) radial function, derived from the boundary conditions of zero radial stress at the outer surface of the coating and continuity of displacement at the coating/substrate interface. The present model instead adopts a hyperbolic-cosine (cosh) radial function, because at the inner interface the radial stress (rather than the displacement) is taken to be continuous, which is more appropriate for an FBG embedded inside a rubber encapsulation whose adhesive layer exhibits finite compliance. (ii) The present cosh-based form yields a closed-form transfer coefficient that is symmetric in r, easier to fit to experimental data, and more directly comparable with the recent multi-layer extension by Zhou et al. (2010) [22].

### 2.1. Strain Transfer Analysis of the Encapsulated FBG

**Figure 2:**
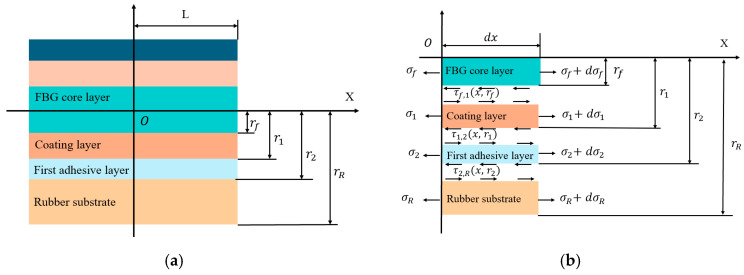
Encapsulated FBG strain transfer model: (**a**) Longitudinal section view of the encapsulated FBG strain transfer model. (**b**) Stress distribution diagram of the encapsulated FBG strain transfer model.

The longitudinal section of the encapsulated FBG strain transfer model was selected as the research object. The total bonding length of the FBG on the rubber substrate is 2L, the center position of the longitudinal section is set as the origin O, and strain transfer calculation is performed for the half-bonding length L (half-length calculation simplifies the derivation process, and the result is applicable to the entire bonding length). As shown in Figure 2, r is the radial distance from the central axis of the FBG to any layer of the model; $r_{f}$, $r_{1}$, $r_{2}$, and $r_{R}$ are the radii of the core layer, coating layer, first adhesive layer, and rubber layer, respectively; $\sigma_{f}$, $\sigma_{1}$, $\sigma_{2}$, and $\sigma_{R}$ are the axial stresses of the core layer, coating layer, first adhesive layer, and rubber layer, respectively; $\tau_{f,1}$, $\tau_{1,2}$, and $\tau_{2,R}$ are the interlayer shear stresses between the core and coating layer, coating layer and first adhesive layer, and first adhesive layer and rubber layer, respectively.

A micro-element dx is randomly taken from the model, and the differential equation of the axial stress $\sigma_{f}$ of the core layer is obtained via the force balance equation:

(2) $$\frac{d\sigma_{f}}{dx}=\frac{2\tau_{f,1}(x,r_{f})}{r_{f}}$$

where $\tau_{f,1}\left(x,r_{f}\right)$ is the shear stress at the interface between the core layer and the coating layer. According to the stress balance relationship of each layer, the shear stress expressions of the coating layer, first adhesive layer, and rubber layer are derived in turn:

(3) $$\tau_{1}\left(x,r\right)=\frac{r_{f}}{r}\tau_{f,1}\left(x,r_{f}\right)+\frac{r^{2}-r_{f}^{2}}{2f}\frac{d\sigma_{1}}{dx}\left(r_{f}<r<r_{1}\right)$$

(4) $$\tau_{2}\left(x,r\right)=\frac{r_{1}}{r}\tau_{1,2}\left(x,r_{1}\right)+\frac{r^{2}-r_{1}^{2}}{2r}\frac{d\sigma_{2}}{dx}\left(r_{1}<r<r_{2}\right)$$

(5) $$\tau_{R}\left(x,r\right)=\frac{r_{2}}{r}\tau_{2,R}\left(x,r_{2}\right)+\frac{r^{2}-r_{2}^{2}}{2r}\frac{dR}{dx}\left(r_{2}<r<r_{R}\right)$$

The outer edge of the rubber layer is a free surface. Substituting the boundary condition $\tau_{R}\left(x,r_{R}\right)=0$ into Formula (5) yields:

(6) $$\frac{dR}{dx}=-\frac{{2r}_{2}}{{r_{R}}^{2}-{r_{2}}^{2}}\tau_{2,R}\left(x,r_{2}\right)$$

Substituting Formula (6) back into Formula (5), the shear stress expression of the rubber layer is simplified as:

(7) $$\tau_{R}\left(x,r_{R}\right)=\frac{r_{2}}{r}(1-\frac{r^{2}-{r_{2}}^{2}}{{r_{R}}^{2}-{r_{2}}^{2}})\tau_{2,R}\left(x,r_{2}\right)$$

Combining Formulas (2)–(7), the following basic assumptions are made for the deformation coordination of each layer: the deformation of the core layer, coating layer, first adhesive layer, and rubber layer occurs simultaneously, and the axial strain differentials of each layer are approximately equal, i.e.,

(8) $$\frac{d\varepsilon_{f}}{dx}≅\frac{d\varepsilon_{1}}{dx}≅\frac{d\varepsilon_{2}}{dx}≅\frac{d\varepsilon_{R}}{dx}$$

According to the stress–strain relationship σ=Eε, the elastic modulus of the core layer is much higher than that of the coating layer and the first adhesive layer, so the following approximate relationships can be derived:

(9) $$\frac{E_{1}}{E_{f}}\frac{d\varepsilon_{1}}{dx}\approx 0,\frac{E_{2}}{E_{f}}\frac{d\varepsilon_{2}}{dx}\approx 0$$

Substituting Formulas (8) and (9) into the shear stress expressions of each layer, and combining the shear modulus formula $G=\frac{E}{2\left(1+\mu \right)}$ (where μ is Poisson’s ratio) and the relationship between interlayer shear stress and shear strain $\tau \left(x,r\right)=G\frac{du}{dr}$ (where u is axial displacement), the second-order linear nonhomogeneous differential equation of the core layer strain is obtained by integrating and simplifying the axial displacement difference between each layer:

(10) $$\frac{d^{2}\varepsilon \left(x,r_{f}\right)}{dx^{2}}+k^{2}\varepsilon \left(x,r_{f}\right)=k^{2}\varepsilon \left(x,r_{R}\right)$$

where $k^{2}$ is the characteristic parameter determined by the material parameters and structural dimensions of each layer, with the expression:

(11) $$k^{2}=2/\left\{{{E_{f}r}_{f}}^{2}\left[\frac{1}{G_{1}}\ln\left(\frac{r_{1}}{r_{f}}\right)+\frac{1}{G_{2}}\ln\left(\frac{r_{2}}{r_{1}}\right)+\frac{1}{G_{R}}[\frac{{r_{R}}^{2}}{{r_{R}}^{2}-{r_{2}}^{2}}\ln\left(\frac{r_{R}}{r_{2}}\right)-\frac{1}{2}]\right]\right\}$$

The two ends of the FBG core layer are free ends of axial stress. Using the boundary conditions $\varepsilon \left(L,r_{f}\right)=\varepsilon \left(-L,r_{f}\right)=0\;$ to solve Formula (10), the strain relationship between the core layer and the rubber layer is obtained:

(12) $$\varepsilon \left(x,r_{f}\right)=\varepsilon \left(x,r_{R}\right)(1-\frac{\cosh\left(kx\right)}{\cosh\left(kL\right)})$$

The strain transfer rate α of the encapsulated FBG is the ratio of the core layer strain to the rubber layer strain, derived as:

(13) $$\alpha =\frac{\varepsilon \left(x,r_{f}\right)}{\varepsilon \left(x,r_{R}\right)}=1-\frac{\cosh\left(kx\right)}{\cosh\left(kL\right)}$$

The average strain transfer rate $\bar{\alpha }$ of the entire FBG bonding section is obtained by integrating the half-bonding length L, reflecting the overall strain transfer efficiency from the rubber substrate to the FBG:

(14) $$\bar{\alpha }=\frac{\bar{\varepsilon \left(x,r_{f}\right)}}{\varepsilon_{r_{R}}}=\frac{2\int_{0}^{L}\varepsilon \left(x,r_{f}\right)dx}{2L\varepsilon_{r_{R}}}=1-\frac{\sinh\left(kL\right)}{kL\cosh\left(kL\right)}$$

Theoretical derivation shows that the strain transfer rate of the encapsulated FBG is mainly related to the FBG bonding length L and the characteristic parameter k. The k value is determined by the shear modulus and thickness of the coating layer, first adhesive layer, and rubber layer. Since the shear modulus and thickness of the coating layer and first adhesive layer are basically fixed during sensor manufacturing, the key factors affecting the strain transfer rate α are the type and thickness of the rubber substrate, as well as the FBG bonding length on the rubber substrate.

### 2.2. Strain Transfer Analysis Between Rubber Substrate and Wind Turbine Blade

**Figure 3:**
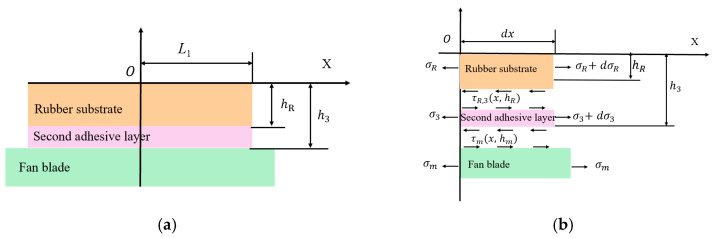
Strain transfer model between rubber substrate and wind turbine blade: (**a**) Longitudinal section view of strain transfer between rubber substrate and wind turbine blade. (**b**) Stress distribution diagram between rubber substrate and wind turbine blade.

The strain transfer model between the rubber substrate and the wind turbine blade takes the half-bonding length $L_{1}$ of the rubber substrate on the wind turbine blade as the research object (the total bonding length is $2L_{1}$). As shown in Figure 3, $h_{R}$, $h_{3}$, and $h_{m}$ are the distances from the origin O to the outermost layers of the rubber layer, second adhesive layer, and wind turbine blade layer, respectively; $\sigma_{R}$, $\sigma_{3}$, and $\sigma_{m}$ are the axial stresses of the rubber layer, second adhesive layer, and wind turbine blade layer, respectively; and $\tau_{R,3}(x,h_{R})$ is the shear stress at the interface between the rubber substrate and the second adhesive layer.

Based on the stress distribution of the model, the differential equation of the axial stress of the rubber layer along the X-axis direction is obtained by force balance analysis:

(15) $$\frac{d\sigma_{R}}{dx}=\frac{\tau_{R,3}\left(x,h_{R}\right)}{h_{R}}$$

Referring to the strain transfer derivation method of the encapsulated FBG, the shear stress expression of the second adhesive layer is derived:

(16) $$\tau_{3}\left(x,h\right)=\tau_{R,3}\left(x,h_{R}\right)+(h-h_{R})\frac{d\sigma_{3}}{dx}(h_{R}<h<h_{3})$$

Substituting Formula (15) into Formula (16) and converting it into the stress–strain relationship according to Hooke’s law σ=Eε:

(17) $$\tau_{3}\left(x,h\right)=E_{R}\frac{d\varepsilon_{R}}{dx}h_{R}+\left(h-h_{R}\right)\frac{d\varepsilon_{3}}{dx}E_{3}$$

It is assumed that the deformation of the rubber layer and the second adhesive layer occurs synchronously, and the axial strain differentials are approximately equal, i.e., $\frac{d\varepsilon_{R}}{dx}≅\frac{d\varepsilon_{3}}{dx}$. Since the elastic modulus of the rubber layer is much lower than that of the second adhesive layer (the difference is an order of magnitude), $\left(E_{R}-E_{3}\right)$ can be simplified to $-E_{3}$ for integral calculation. By integrating and simplifying the axial strain difference between the rubber layer and the second adhesive layer, the second-order linear nonhomogeneous differential equation of the rubber layer strain is obtained:

(18) $$\frac{d^{2}\varepsilon_{R}}{{dx}^{2}}+{k_{1}}^{2}\varepsilon \left(x,h_{R}\right)={k_{1}}^{2}\varepsilon \left(x,h_{3}\right)$$

where $k_{1}^{2}$ is the characteristic parameter of this stage, determined by the material parameters and structural dimensions of the rubber layer and the second adhesive layer:

(19) $${k_{1}}^{2}=\frac{G_{3}}{E_{3}[\frac{\left({h_{3}}^{2}+{h_{R}}^{2}\right)}{2}-h_{3}h_{R}]}$$

Using the boundary conditions to solve Formula (18), the strain transfer rate β between the rubber substrate and the wind turbine blade is derived as:

(20) $$\beta =1-\frac{\cosh\left(k_{1}x\right)}{\cosh\left(k_{1}L_{1}\right)}$$

The average strain transfer rate $\bar{\beta }$ of the entire rubber substrate bonding section is obtained via integral calculation, which reflects the overall strain transfer efficiency from the wind turbine blade to the rubber substrate:

(21) $$\bar{\beta }=1-\frac{\sinh\left(k_{1}L_{1}\right)}{k_{1}L_{1}\cosh\left(k_{1}L_{1}\right)}$$

Theoretical derivation shows that the strain transfer rate β is mainly affected by the thickness and elastic modulus of the second adhesive layer, as well as the thickness and bonding length of the rubber substrate. Among these, the thickness and bonding length of the rubber substrate are the key adjustable structural parameters in sensor design, providing a theoretical basis for the structural optimization of rubber-encapsulated FBG sensors.

## 3. Analysis of Factors Affecting Strain Transfer

Based on the established strain transfer model, the factors affecting the strain transfer efficiency of rubber-encapsulated FBG sensors include rubber substrate thickness, FBG bonding length, rubber substrate bonding length, rubber substrate material, and FBG material parameters. Through the performance comparison of various industrial rubbers, the ethylene propylene diene monomer (EPDM) rubber is selected as the sensor’s encapsulation material for wind turbine blade monitoring due to its excellent weather resistance, aging resistance, elasticity, and processing performance. The material parameters of each layer of the strain transfer model are shown in Table 1. In practical sensor applications, the bonding length of the rubber substrate should be moderate: an excessively long bonding length will lead to unclear measuring point positions, while an excessively short bonding length will reduce strain transfer efficiency. Therefore, the total length of the rubber sensor substrate is tentatively set to 11 cm, and the rubber sensor is fully bonded to the wind turbine blade surface, resulting in a half-bonding length $L_{1}$ of 55 mm for the rubber substrate. The control variable method is used to analyze the two most critical adjustable parameters (FBG bonding length and rubber substrate thickness) on strain transfer efficiency, providing a theoretical basis for the sensor’s structural dimensions.

### 3.1. Effect of FBG Bonding Length

**Figure 4:**
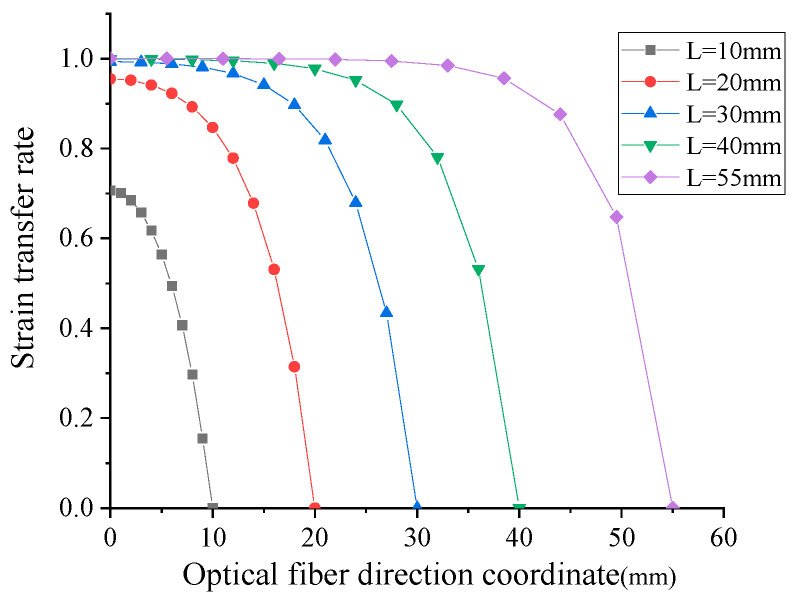
Relationship between strain transfer efficiency and FBG bonding length.

To investigate the influence of FBG bonding length on the strain transfer efficiency, the control variable method was adopted, and the dimensions of the rubber substrate were first determined. The EPDM rubber substrate with a thickness of 4 mm, a width of 1.5 cm, and a total length of 11 cm was selected. The half-bonding lengths of the FBG were set to 10 mm, 20 mm, 30 mm, 40 mm, and 55 mm, respectively, and the corresponding strain transfer curves were calculated for each case. Each curve contained ten calculation points, which were the ten equal division points of the half-bonding length. Figure 4 shows the relationship between strain transfer efficiency and FBG bonding length. All five curves generally exhibited a saturating behavior, and the strain transfer rate decreased more rapidly as it approached the terminal end of the bonding area. With the increase in the half-bonding length, the strain transfer at each position was improved. Therefore, increasing the half-bonding length of the fiber as much as possible can improve the average strain transfer rate. When L = 10 mm, the maximum strain transfer rate at the central position was only about 70%. As the coordinate increased, the strain transfer rate decreased. When L = 20 mm, the maximum strain transfer rate at the central position increased to 95%, and it could reach 90% within the first 10 mm. When L = 30 mm, 40 mm, and 55 mm, the maximum strain transfer rate at the central position could reach 99%, and the average strain transfer rate could all exceed 80%, which basically met the requirements of strain monitoring for wind turbine blades. Therefore, the total bonding length of the fiber should not be less than 60 mm.

### 3.2. Effect of Rubber Substrate Thickness

**Figure 5:**
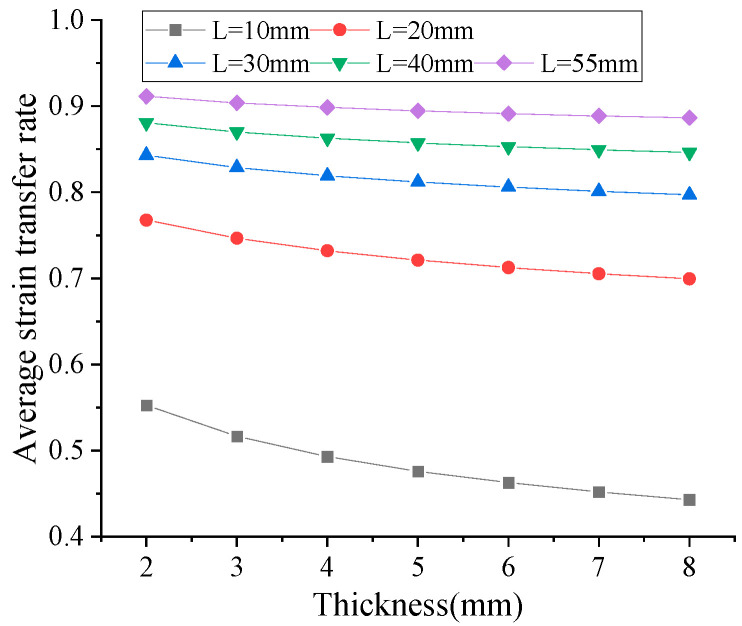
Relationship between average strain transfer efficiency and rubber substrate thickness.

To explore the combined effect of rubber substrate thickness and FBG bonding length on strain transfer efficiency, the average strain transfer efficiencies of sensors with rubber substrate thicknesses of 2 mm, 3 mm, 4 mm, 5 mm, 6 mm, 7 mm, and 8 mm were calculated for five FBG half-bonding lengths (10 mm, 20 mm, 30 mm, 40 mm, and 55 mm). Figure 5 shows the relationship between the average strain transfer efficiency of the sensor and the substrate thickness.

The calculation results reveal two key rules: ① Under the same FBG bonding length, the average strain transfer efficiency of the sensor decreases with the increase in rubber substrate thickness. The increase in rubber substrate thickness leads to an increase in shear deformation loss during strain transfer, resulting in reduced strain transfer efficiency. ② The increase in FBG bonding length weakens the influence of rubber substrate thickness on the strain transfer efficiency. The longer the FBG bonding length, the slower the decline rate of the average strain transfer efficiency with the increase in rubber substrate thickness. When the FBG half-bonding length is greater than 40 mm, the change in rubber substrate thickness has a slight effect on the average strain transfer efficiency.

From an engineering application perspective, the rubber substrate thickness cannot be too small: an excessively thin substrate will cause the armor wire at the sensor end to crack and fall off, seriously affecting the service life of the sensor. An armor wire with an outer diameter of 3 mm can meet the tensile strength required for sensor encapsulation. Combined with the sensor manufacturing process, the sensor end is prone to damage in practical applications when the rubber substrate thickness is less than 4 mm, while the strain transfer efficiency decreases significantly when the thickness exceeds 4 mm. Therefore, considering both strain transfer efficiency and sensor service life, 4 mm is determined as the optimal thickness of the EPDM rubber substrate.

## 4. Strain Transfer Test of Sensors with Different Substrate Thickness

### 4.1. Test Preparation

**Figure 6:**
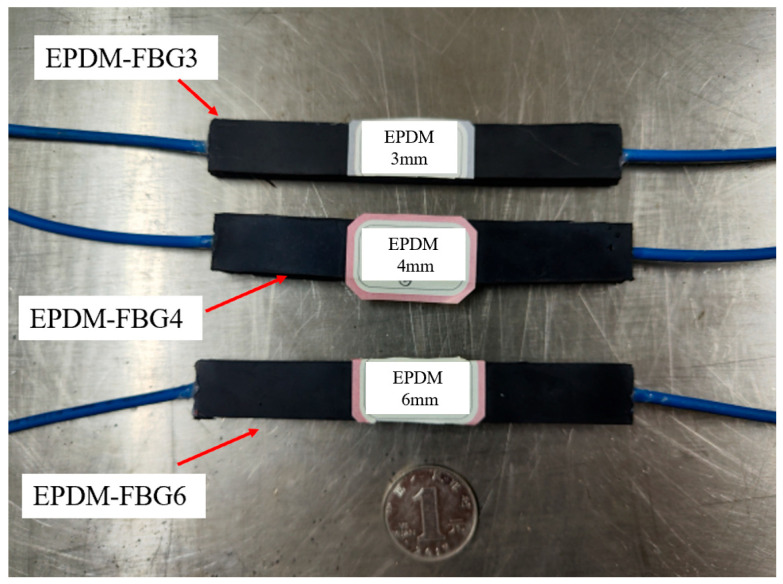
EPDM-encapsulated FBG sensors with rubber substrate thicknesses of 3 mm, 4 mm, and 6 mm.

To further verify the correctness of the established strain transfer model for rubber-encapsulated FBG sensors and the conclusions of the influencing factor analysis, strain transfer tests were conducted on sensors with different EPDM rubber substrate thicknesses. Three types of EPDM rubber-encapsulated FBG sensors with substrate thicknesses of 3 mm, 4 mm, and 6 mm were fabricated, as shown in Figure 6, and named EPDM-FBG3, EPDM-FBG4, and EPDM-FBG6, respectively. The other structural parameters of the three sensors were kept consistent to eliminate the influence of other factors on the test results: a width of 1.5 cm, a total length of the substrate and protective layer of 11 cm, a total FBG bonding length of 10 cm, and a protective layer rubber thickness of 2 mm. The FBG, with a grating length of 10 mm and a reflectivity greater than 90%, was purchased from Dalian Boruixin Technology Co., Ltd., Dalian, China. The strain sensitivity coefficients of the three EPDM rubber-encapsulated FBG sensors were approximately 2.00 pm/με.

**Figure 7:**
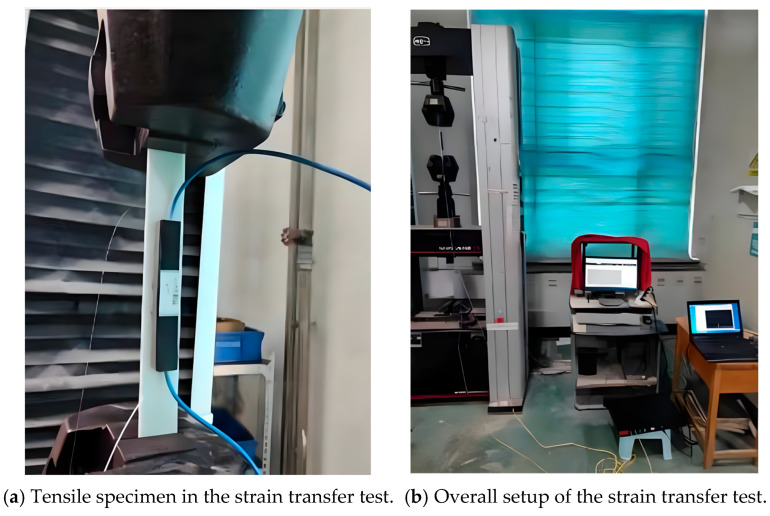
Test setup.

To better simulate the strain transfer characteristics in the actual wind turbine blade monitoring environment, fiber-reinforced plastic (FRP) strips with surface properties similar to those of wind turbine blades were used as the tested substrate in this test. The specimen dimensions were 30 cm in length, 3 mm in thickness, and 3 cm in width. In each test, an EPDM rubber-encapsulated sensor was bonded to the center of the FRP plate with a total bonding length of 11 cm, and a bare FBG sensor was bonded at the same position, with its grating region strictly aligned with the center of the rubber-encapsulated sensor and the same total bonding length of 11 cm. During the test, the strain measured by the bare FBG sensor was used as the reference true strain of the FRP plate. Figure 7 shows the schematic diagram of the strain transfer test specimen and the test setup. A tensile testing machine was used to apply tensile loading to the FRP plate, and an FBG demodulator was employed to synchronously collect the central wavelength data of both the EPDM rubber-encapsulated sensor and the bare FBG sensor. The measured strain values were converted using the respective strain sensitivity coefficients of the two sensors, and the strain transfer efficiency of the encapsulated sensor was finally obtained by comparing the measured strains of the two sensors. Since the indoor ambient temperature remained nearly constant during the test, the effect of temperature on strain measurement was negligible, and no temperature compensation was required. The test process was as follows: the tensile force was gradually increased from 0 kN to 5 kN with a load increment of 0.5 kN per step, and each load step was maintained for 3 min to ensure that the strains of the FRP plate and the sensors reached a stable state. During each load step, the FBG demodulator continuously recorded the central wavelength values of the EPDM rubber-encapsulated sensor and the bare FBG sensor.

Let the strain of the bonding section measured by the rubber-encapsulated sensor be A, and the average strain of the bonding section measured by the bare FBG sensor be B. The measured strain transfer efficiency γ of the rubber-encapsulated sensor is calculated by the following formula:

(22) $$\gamma =\frac{A}{B}$$

The measured strain transfer efficiency of the sensor under each load level was calculated according to Formula (22), and the theoretical strain transfer efficiency was calculated based on the strain transfer model established in this study, using the material parameters in Table 1 and the actual structural dimensions of the sensor. The test results were compared with the theoretical results to calculate the relative error (relative error = |measured value—theoretical value|/theoretical value × 100%), verifying the validity and accuracy of the established strain transfer model.

### 4.2. Test Results and Analysis

Strain transfer tensile tests were conducted on the three EPDM-FBG sensors, and Table 2 shows the initial central wavelengths of the three sensors and the three bare FBGs for comparison.

**Figure 8:**
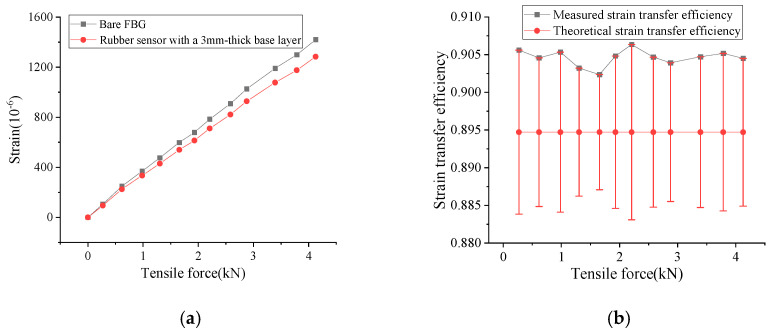
Tensile test results for EPDM-FBG3. (**a**) Relationship between strain and tensile force. (**b**) Comparison of theoretical and measured strain transfer efficiency.

**Figure 9:**
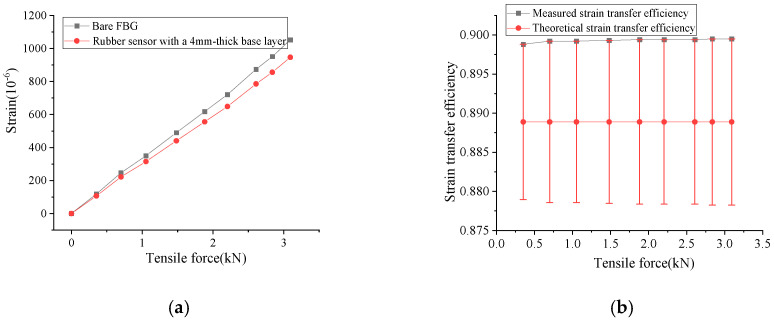
Tensile test results for EPDM-FBG4. (**a**) Relationship between strain and tensile force. (**b**) Comparison of theoretical and measured strain transfer efficiency.

**Figure 10:**
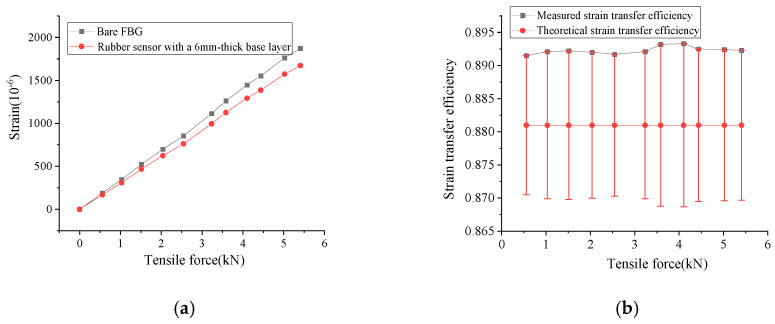
Tensile test results for EPDM-FBG6. (**a**) Relationship between strain and tensile force. (**b**) Comparison of theoretical and measured strain transfer efficiency.

Figure 8, Figure 9 and Figure 10 show the tensile test results. For the EPDM-FBG3 sensor, the theoretical strain transfer efficiency calculated by the established model is 0.8947, and the average measured strain transfer efficiency under each tensile load level (0.5–5 kN) is 0.9046, with a relative error of 1.11% compared with the theoretical value. For the EPDM-FBG4 sensor, the theoretical strain transfer efficiency is 0.8889, and the average measured strain transfer efficiency under each tensile load level is 0.8993, with a relative error of 1.17% compared with the theoretical value. For the EPDM-FBG6 sensor, the theoretical strain transfer efficiency is 0.8810, and the average measured strain transfer efficiency under each tensile load level is 0.8923, with a relative error of 1.28% compared with the theoretical value. The test results show that the measured strain transfer efficiencies of the three EPDM-FBG sensors with different substrate thicknesses all decrease with the increase in rubber substrate thickness, which is completely consistent with the theoretical analysis conclusion.

The test results also verify the conclusion of the optimal structural parameters of the sensor obtained from theoretical analysis: the EPDM rubber substrate with a thickness of 4 mm balances both strain transfer efficiency and sensor service life, with a measured strain transfer efficiency of 0.8993 (close to that of the 3 mm-thick sensor) and no end damage during repeated tests. In contrast, the EPDM-FBG3 sensor has a slightly higher strain transfer efficiency but shows slight cracks at the end armor wire after repeated tests, which may affect its service life in long-term engineering applications. The EPDM-FBG6 sensor has no end damage but a significantly lower strain transfer efficiency (0.8923), which is still within the engineering accuracy requirement but not optimal. The total FBG bonding length is 10 cm (≥60 mm), and the average strain transfer efficiency of the sensor exceeds 80%, ensuring the strain transfer efficiency of the sensor and verifying the rationality of the structural parameter optimization.

## 5. Model Test for Strain Monitoring of Wind Turbine Blades Using EPDM-FBG Strain Rosettes

Historical context of FBG-rosette strain sensors. Multi-axis strain measurement using FBG rosettes has been investigated for more than two decades. Magne et al. (1997) [23] first demonstrated a 2-axis (0°/90°) in-fiber Bragg grating rosette for transverse strain discrimination, while Haran et al. (1998) [24] extended the concept to a 3-element (0°/45°/90°) rosette for full in-plane strain tensor reconstruction on composite panels. The present work adopts the same 0°/45°/90° configuration, but unlike the earlier work that used bare or metal-coated FBGs.

### 5.1. Development of EPDM-FBG Strain Rosettes

FBG technology has been widely applied in wind turbine blade strain monitoring, and most of these applications focus on unidirectional strain monitoring along the sensor orientation. Wind turbine blades bear complex mechanical loads during operation, including their own gravity, centrifugal force, and aerodynamic force. The direction of the maximum strain is uncertain and may not align with the sensor orientation in some cases. In addition, variable pitch technology is commonly adopted for high-power wind turbines, and pitch regulation under high wind speeds is prone to excessive strain that may damage the weak points of the blades. Therefore, it is necessary to monitor the blade strain during pitch regulation. During the pitch rotation process, the strain on the surface of wind turbine blades is not unidirectional but belongs to two-dimensional plane strain. Deploying sensors in a single direction cannot effectively obtain the magnitude and direction of the principal stresses, failing to meet the requirements of strain monitoring. Based on this, an EPDM-FBG strain rosette sensor was designed in this study to monitor the direction and magnitude of the principal strains of wind turbine blades. For plane strain analysis, there are three strain components on the blade surface: $\varepsilon_{x}$, $\varepsilon_{y}$ and $\gamma_{xy}$. Assuming these three components are known, the values of the principal strain $\varepsilon_{\alpha }$ and shear strain $\gamma_{\alpha }$ at an angle α between the measuring point and the $\varepsilon_{x}$ direction can be obtained as follows:

(23) $$\varepsilon_{\alpha }=\frac{\varepsilon_{x}+\varepsilon_{y}}{2}+\frac{\varepsilon_{x}-\varepsilon_{y}}{2}\cos2\alpha -\frac{\gamma_{xy}}{2}\sin2\alpha$$

(24) $$\frac{\gamma_{\alpha }}{2}=\frac{\varepsilon_{x}-\varepsilon_{y}}{2}\sin2\alpha +\frac{\gamma_{xy}}{2}\cos2\alpha$$

In the direction of the principal strain, the shear strain is zero, so Equation (24) can be simplified to:

(25) $$tan2\alpha_{0}=\frac{-\gamma_{xy}}{\varepsilon_{x}-\varepsilon_{y}}=\;\frac{\varepsilon_{0^{0}}+\varepsilon_{90^{0}}-2\varepsilon_{45^{0}}}{\varepsilon_{0^{0}}-\varepsilon_{90^{0}}}$$

The principal strain direction $\alpha_{0}$ is defined as the angle (measured counter-clockwise from the 0-degree FBG axis) at which the shear strain component $\gamma_{xy}$ vanishes and the normal strain component reaches its extremum.

However, the three strain components $\varepsilon_{x}$, $\varepsilon_{y}$ and $\gamma_{xy}$ cannot be directly measured in practical applications. Therefore, it is necessary to first measure the linear strains $\varepsilon_{\alpha 1}$, $\varepsilon_{\alpha 2}$ and $\varepsilon_{\alpha 3}$ in any three directions $\alpha_{1}$, $\alpha_{2}$ and $\alpha_{3}$, and substitute them into Equation (23):

(26) $$\varepsilon_{\alpha 1}=\frac{\varepsilon_{x}+\varepsilon_{y}}{2}+\frac{\varepsilon_{x}-\varepsilon_{y}}{2}\cos2\alpha_{1}-\frac{\gamma_{xy}}{2}\sin2\alpha_{1}$$

(27) $$\varepsilon_{\alpha 2}=\frac{\varepsilon_{x}+\varepsilon_{y}}{2}+\frac{\varepsilon_{x}-\varepsilon_{y}}{2}\cos2\alpha_{2}-\frac{\gamma_{xy}}{2}\sin2\alpha_{2}$$

(28) $$\varepsilon_{\alpha 3}=\frac{\varepsilon_{x}+\varepsilon_{y}}{2}+\frac{\varepsilon_{x}-\varepsilon_{y}}{2}\cos2\alpha_{3}-\frac{\gamma_{xy}}{2}\sin2\alpha_{3}$$

Since $\varepsilon_{\alpha 1}$, $\varepsilon_{\alpha 2}$ and $\varepsilon_{\alpha 3}$ are all known quantities, the strain components $\varepsilon_{x}$, $\varepsilon_{y}$ and $\gamma_{xy}$ can be solved by substituting the measured values into the above equations. The direction of the principal strain $\alpha_{0}$ can be obtained from Equation (25), and the maximum and minimum principal strain values can be calculated by substituting $\alpha_{0}$ into Equation (23):

(29) $$\varepsilon_{max}=\frac{\varepsilon_{x}+\varepsilon_{y}}{2}+\sqrt{{\left(\frac{\varepsilon_{x}-\varepsilon_{y}}{2}\right)}^{2}+{\left(\frac{\gamma_{xy}}{2}\right)}^{2}}$$

(30) $$\varepsilon_{min}=\frac{\varepsilon_{x}+\varepsilon_{y}}{2}-\sqrt{{\left(\frac{\varepsilon_{x}-\varepsilon_{y}}{2}\right)}^{2}+{\left(\frac{\gamma_{xy}}{2}\right)}^{2}}$$

**Figure 11:**
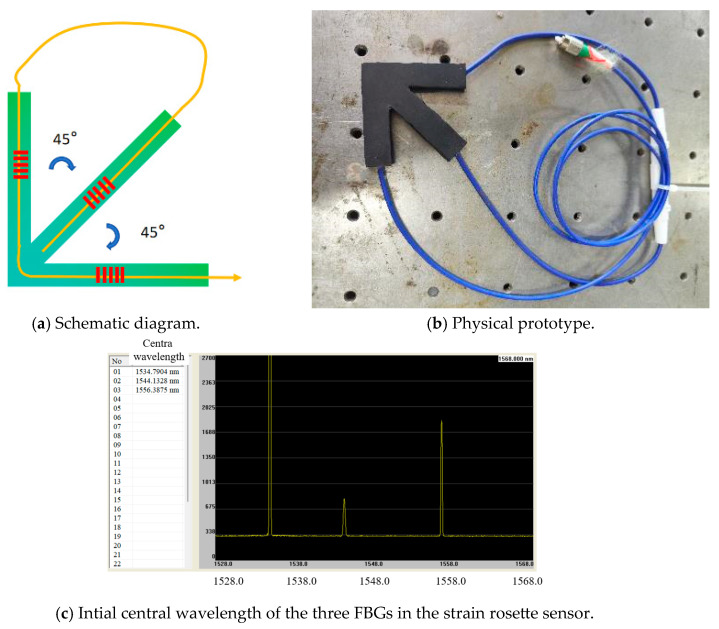
Structural schematic and physical prototype of the EPDM-FBG strain rosette sensor.

In practical monitoring, $\alpha_{1}$, $\alpha_{2}$ and $\alpha_{3}$ are set to values that facilitate calculation. The EPDM-FBG strain rosette sensor designed in this study adopts a right-angle strain rosette configuration, where ${\;\alpha }_{1}=0{}^{\circ},\;\alpha_{2}=45{}^{\circ}$ and $\alpha_{3}=90{}^{\circ}$. Figure 11 shows the structural schematic and physical prototype of the EPDM-FBG strain rosette sensor. The rubber substrate is cut into the shape as shown in the figure, with each directional branch having a width of 1.5 cm, a length of 11 cm and a thickness of 4 mm. Based on the wavelength division multiplexing (WDM) principle, three FBGs with different central wavelengths are connected in series on a single optical fiber, and a rubber protective sheet of the same shape with a thickness of 2 mm is bonded on the substrate. The minimum bend radius of the polyimide-coated fiber Bragg grating (FBG) adopted in this study is 10 mm. Therefore, during sensor fabrication, the bend radius of all three FBGs encapsulated inside the rubber substrate is required to exceed 10 mm. The initial central wavelengths of three FBGs in the strain rosette sensor are 1534.7904 nm, 1544.1328 nm and 1556.3875 nm respectively.

Substituting α1 = 0°, α2 = 45° and α3 = 90° into Equations (26)–(28) yields the following results:

(31) $$\varepsilon_{max}=\frac{\varepsilon_{0{}^{\circ}}+\varepsilon_{90{}^{\circ}}}{2}+\sqrt{{\left(\frac{\varepsilon_{0{}^{\circ}}-\varepsilon_{90{}^{\circ}}}{2}\right)}^{2}+{\left(\frac{\varepsilon_{0{}^{\circ}}+\varepsilon_{90{}^{\circ}}-2\varepsilon_{45{}^{\circ}}}{2}\right)}^{2}}$$

(32) $$\varepsilon_{min}=\frac{\varepsilon_{0{}^{\circ}}+\varepsilon_{90{}^{\circ}}}{2}-\sqrt{{\left(\frac{\varepsilon_{0{}^{\circ}}-\varepsilon_{90{}^{\circ}}}{2}\right)}^{2}+{\left(\frac{\varepsilon_{0{}^{\circ}}+\varepsilon_{90{}^{\circ}}-2\varepsilon_{45{}^{\circ}}}{2}\right)}^{2}}$$

### 5.2. Model Test for Strain Monitoring of Wind Turbine Blades

A model test for strain monitoring of wind turbine blades using the EPDM-FBG strain rosette was carried out in this section. The EPDM-FBG strain rosette was deployed on the irregular curved surface at the root of the windward side of a small-scale wind turbine blade model, where the strain distribution of the blade is relatively complex.

**Figure 12:**
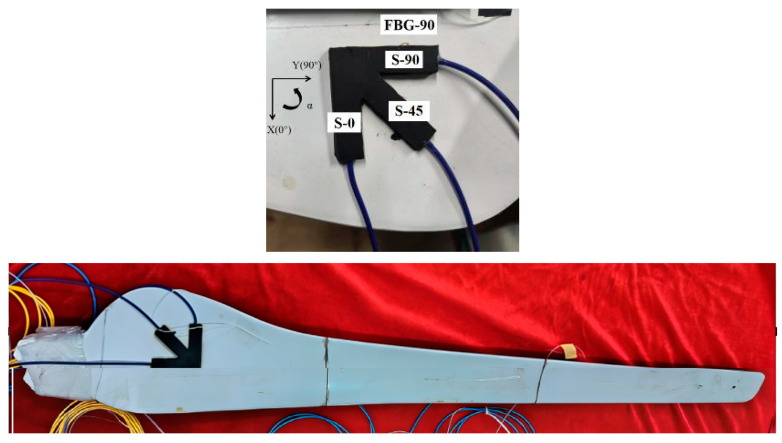
Layout of the rubber-encapsulated FBG right-angle strain rosette sensor.

**Figure 13:**
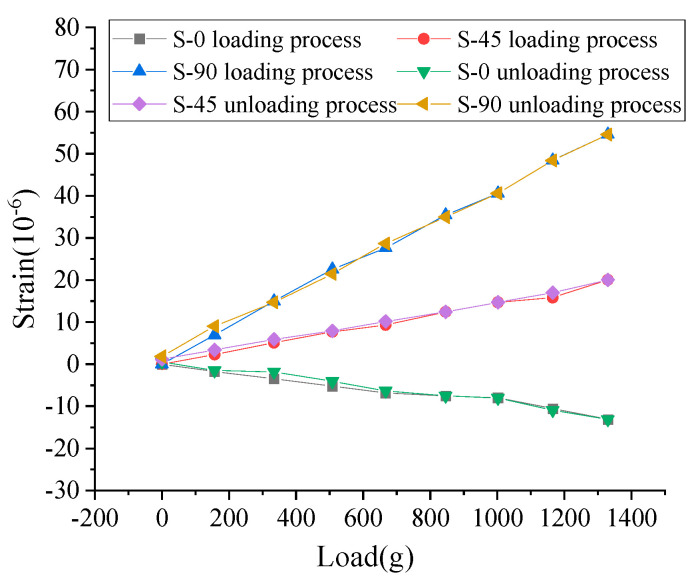
Blade surface strain values measured by the S-0, S-45 and S-90 branches of the strain rosette sensor.

A vertical static load test was conducted to verify the performance of the EPDM-FBG strain rosette sensor in monitoring the principal strains of the wind turbine blade. Figure 12 shows the layout of the strain rosette sensor. The vertically downward direction is defined as the X direction (corresponding to an angle α of 0°), and the counterclockwise direction is set as the positive direction. The sensor branches in the 0°, 45°, and 90° directions are named S-0, S-45, and S-90, respectively. A reference FBG sensor along the Y-axis direction is deployed directly above the strain rosette and named FBG-90. The strain rosette sensor is located 14 cm from the blade root and below the center line of the windward side. The size and fabrication process of each branch sensor are completely consistent with those of the ordinary EPDM-FBG sensor, with a rubber substrate thickness of 4 mm. Therefore, its average strain transfer efficiency and strain sensitivity are the same as those of the ordinary rubber-encapsulated sensor. The loading point is located 85 cm from the blade root. The measured strain values were calculated from the central wavelength of the strain rosette sensor. Subsequently, based on the strain transfer analysis theory, the actual surface strain values of the blade measured by the strain rosette sensor were computed using Formula (22). Figure 13 presents the actual surface strain values of the blade measured by the strain rosette sensor branches S-0, S-45, and S-90.

**Figure 14:**
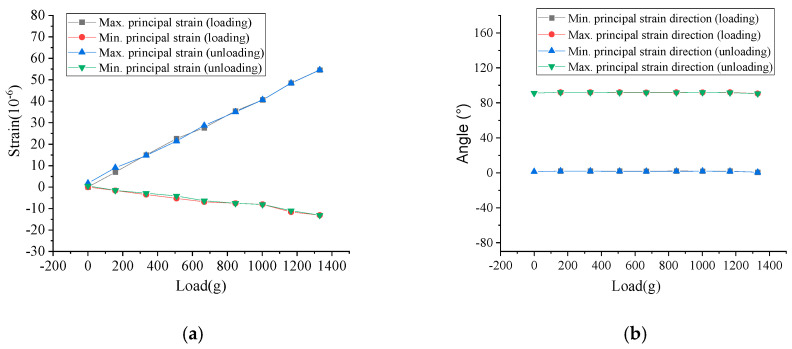
Magnitudes and directions of the maximum and minimum principal strains: (**a**) Magnitudes of the maximum and minimum principal strains. (**b**) Directions of the maximum and minimum principal strains.

The strain values of S-0, S-45, and S-90 under each load step were substituted into Formulas (31)–(32) to calculate the magnitudes and directions of the maximum and minimum principal strains during the vertical loading process. Figure 14 shows the magnitudes and directions of the maximum and minimum principal strains.

**Figure 15:**
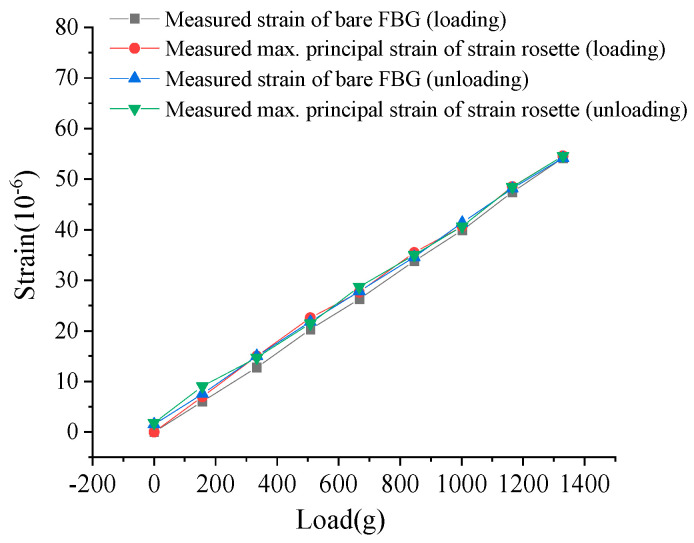
Comparison of the maximum principal strain measured by the strain rosette and the strain measured by the bare FBG (FBG-90).

The linearity of the fitted straight lines for the maximum principal strain during the loading and unloading processes was 0.9986 and 0.9989, respectively, while the linearity of the fitted straight lines for the minimum principal strain during the same processes was 0.9732 and 0.9828. All linearity values are higher than 0.97, indicating good linearity and demonstrating that the strain rosette sensor possesses excellent strain-sensing characteristics. Comparative analysis shows that the maximum principal strain curve basically coincides with the strain curve of S-90, and the minimum principal strain curve basically coincides with the strain curve of S-0. This indicates that under vertical loads, the maximum principal strain is almost distributed along the axial direction of the wind turbine blade, and the minimum principal strain is almost distributed along the direction perpendicular to the blade axis. As can be seen from Figure 14b, the average direction of the maximum principal strain under each load step forms an angle of 91.75° with the X-axis (almost coinciding with the blade axis), and the average direction of the minimum principal strain forms an angle of 1.75° with the X-axis (almost coinciding with the X-axis), which is consistent with the aforementioned conclusions. To further verify the accuracy of the strain rosette test results, the maximum principal strain of the blade measured by the strain rosette was compared with the strain measured by the FBG-90 sensor (arranged along the axial direction of the wind turbine blade), as shown in Figure 15. The maximum principal strain curve is in good agreement with the measured strain curve of FBG-90, which proves that the EPDM rubber-encapsulated right-angle strain rosette sensor can effectively monitor the direction and magnitude of the principal strains on the blade surface and meet the monitoring requirements in practical engineering applications.

## 6. Conclusions

To address the strain transfer deviation of rubber-encapsulated FBG (FBG) sensors in wind turbine blade monitoring, this study establishes a surface-bonded strain transfer model. Combined with experimental validation and parametric analysis, the main conclusions are summarized as follows:

(1) The established surface-bonded strain transfer model can accurately characterize the strain transfer law of rubber-encapsulated FBG sensors. The overall strain transfer efficiency is defined as the product of the transfer efficiency inside the encapsulated FBG and that at the rubber–blade interface. The relative error between model predictions and experimental measurements is less than 2%, which verifies the high precision and reliability of the proposed model.
(2) Structural parameters significantly affect the strain transfer performance of sensors. The FBG bonding length is positively correlated with strain transfer efficiency, and an efficiency exceeding 80% can be achieved when the bonding length is more than 60 mm. In contrast, rubber substrate thickness presents a negative correlation with strain transfer efficiency, and this adverse effect can be effectively weakened by increasing the bonding length. Considering sensing performance and service durability comprehensively, 4 mm is determined as the optimal thickness of the EPDM rubber substrate.
(3) The EPDM-encapsulated FBG sensor possesses excellent static measurement performance. The variation in rubber substrate thickness has a negligible effect on sensor strain sensitivity. The developed right-angle FBG strain rosette can precisely capture the complex plane strain states of wind turbine blades, with the linearity of principal strain tests higher than 0.97, satisfying the requirements of practical engineering monitoring.

This study clarifies the optimal structural parameters of rubber-encapsulated FBG sensors, providing theoretical guidance for their structural design and engineering application in wind turbine blade strain monitoring. Future research will focus on the influences of dynamic loads and environmental factors (e.g., temperature and humidity) on strain transfer performance. A temperature-humidity compensation model will be established to improve the environmental adaptability of the sensor. Furthermore, finite element simulation can be adopted to conduct multi-parameter structural optimization and further enhance the strain transfer efficiency of the sensor. Furthermore, while the present study focuses on the strain transfer from the host structure to the FBG and on the reconstruction of the in-plane strain components, the next step is the conversion from the measured strain tensor to the stress tensor via the plane-stress constitutive relation.

## Figures and Tables

**Table 1:** Material parameters of the strain transfer model.

Structural Layer	Dimension (mm)	Elastic Modulus (GPa)	Poisson’s Ratio
Core layer	Radius: 0.0625	72	0.17
Coating layer	Outer diameter: 0.125	3	0.35
First adhesive layer	Thickness: 0.1	4	0.35
EPDM rubber layer	Thickness: 2~8	0.036	0.45
Second adhesive layer	Thickness: 0.3	4	0.35

**Table 2:** Initial central wavelengths of the EPDM-FBG sensors and bare FBGs (nm).

Test 1		Test 2		Test 3	
EPDM-FBG3	Bare FBG	EPDM-FBG4	Bare FBG	EPDM-FBG6	Bare FBG
1556.9848	1551.2150	1544.4232	1545.7873	1533.0724	1545.7873

## Data Availability

The data presented in this study are available upon request from the corresponding author.
